# Targeting the secretory program of 3q-amplified lung cancers

**DOI:** 10.1172/JCI181798

**Published:** 2024-06-17

**Authors:** Luis Pardo, Jim C. Norman

**Affiliations:** 1 Cancer Research UK Scotland Institute, Glasgow, United Kingdom.; 2 School of Cancer Sciences, University of Glasgow, Glasgow, United Kingdom.

## Abstract

Designing strategies to target cell proliferation has been a priority of cancer researchers for decades. However, targeting the secretory programs of transformed cells can influence other cancer features such as cell survival, migration, and communication with the tumor stroma. In this issue of the *JCI*, Tan and colleagues describe functional cooperativity between the Golgi-resident proteins Golgi integral membrane protein 4 (GOLIM4) and ATPase secretory pathway Ca2^+^ transporting 1 (ATP2C1) in the coordination of a secretory program in 3q-amplified cancers. Targeting these tumors with manganese (Mn^2+^) promoted GOLIM4 degradation and imposed a secretory blockade that impaired tumor progression and stromal cell recruitment in mice. These findings highlight the secretory program as a therapeutic target in 3q-amplified malignancies and provide a promising strategy to treat tumor progression.

## Genomic amplicons target vesicle trafficking in cancer

Many cancers are characterized by increased copies of genome regions (known as amplicons) containing genes that drive tumor growth and aggressiveness. Identification of the driver genes in amplicons is critical for developing approaches to treat cancer, as they can represent targets at which to aim anticancer drugs. Moreover, because genes are often physically clustered according to their function, an amplicon likely comprises several genes, which, together, support a cellular function that can drive cancer. For instance, 17q12 is a well-documented cancer amplicon harboring genes that drive signaling pathways promoting cancer cell proliferation and survival (1). These gene products, notably the HER2 RTK, are now established targets for cancer therapy. Other amplicons, including those comprising genes for Rab GTPases (1q22, which contains RAB25, and 6p11, containing RAB23), drive cancer by impinging on vesicle trafficking (2, 3). Indeed, Rab GTPases are key regulators of the endomembrane transport, and the Rab 11 effector Rab-coupling protein (RCP) (also known as RAB11FIP1), drives the 8p11-12 amplicon, which is associated with poor cancer outcomes (4). Subsequent studies showed that RCP and RAB25 play important roles in regulating intracellular trafficking of integrins and RTKs, including EphA2 and EGFR1, in ways that support cancer cell migration and invasiveness (5, 6). More recently, it was reported that specific targeting of RCP in pancreatic adenocarcinoma opposes mutant p53–driven metastasis to the liver and lungs (7).

## Trafficking through the biosynthetic pathway in cancer

The biosynthetic secretory pathway is also emerging as a membrane-trafficking process that is dysregulated in cancer. Genetic damage can drive secretory programs, and perhaps the most well-characterized example is the senescence-associated secretory phenotype (SASP). The SASP can be driven by upregulated MAPK signaling following acquisition of activating mutations in proto-oncogenes such as Ras and Raf and by other potentially carcinogenic events such as genetic instability (8). Upregulated synthesis of numerous proteins including soluble intercellular signaling factors (i.e., chemokines and growth factors) and large, insoluble extracellular matrix (ECM) components characterizes the secretome accompanying oncogene-induced senescence. Importantly, SASP production is accompanied by a massively increased secretory capacity characterized by expansion of the Golgi complex and vesicle transport machinery. Functionally, the SASP is thought to recruit myeloid and lymphoid cells to mediate immune clearance of mutated cells, thus constituting a tumor surveillance/suppression mechanism (9–11). Furthermore, in certain tissues, such as the liver, insoluble SASP factors likely contribute to regenerative niches that encourage replacement of damaged and mutated cells. However, the recruitment of immune cells, particularly myeloid cells, and the generation of ECM-based regenerative niches can contribute to microenvironments favorable to tumor growth (12). Consistently, hypersecretory programs that are now being linked to oncogenesis and tumor progression share many common features with the SASP, including the copious release of inflammatory mediators and factors that promote cell migration and upregulated flux through the ER and Golgi complex (8, 12).

Characterization of hypersecretory phenotypes in cancer may identify valuable biomarkers for early detection and reveal new therapeutic targets. Moreover, because there is overlap between cancer hypersecretory phenotypes and the SASP, it is critical that we understand the secretory mechanisms invoked by established cancers whose secretomes are more likely to be protumorigenic. Chromosomal instability and the acquisition of amplicons that drive disease progression are relatively late events in tumorigenesis. Therefore, identification of the drivers of hypersecretory phenotypes within amplicons associated with poor cancer outcomes has become a priority. Furthermore, cancer-secreted factors may cause different effects in tumor and stromal cells, making it difficult to assess whether a specific secreted protein will play pro- or antitumorigenic roles. In addition, compensatory effects due to inhibition of a single protein or pathway may alter the tumor response and lead to therapy resistance. It is therefore important to explore more deeply the integrative therapies that target complex tumor secretomes driven by particular cancer genotypes, including those associated with cancer amplicons. In this issue of the *JCI*, Tan et al. explored the cancer-amplified chromosomal region 3q. The authors uncovered a functional cooperation between two Golgi-resident proteins in the coordination of a secretory program implicated in cancer cell survival and tumor microenvironment (TME) activation (13).

## GOLIM4 activates a secretory program in 3q-amplified cancers

Tan and authors have previously described a malignant secretory program that is activated as epithelial cancer cells undergo transition to a more mesenchymal phenotype and is regulated by Golgi-resident proteins (14). Their most recent work reveals how Golgi-integral membrane protein 4 (GOLIM4) participates in the secretion of a plethora of protumorigenic proteins in 3q-amplified cells. Using a combination of in vitro (e.g., anchorage-independent growth and migratory/invasive behavior) and in vivo (syngeneic and xenograft models) approaches, the authors demonstrated the role of these secretory programs in supporting tumor survival and progression. Various mechanistic aspects of GOLIM4-regulated secretion were explored, including the protein’s role in cargo sorting through interaction with ATPase secretory pathway Ca2^+^ transporting 1 (ATP2C1) and its role in vesicle formation, and budding through interaction with Golgi phosphoprotein 3 (GOLPH3) (13).

**Figure 1:**
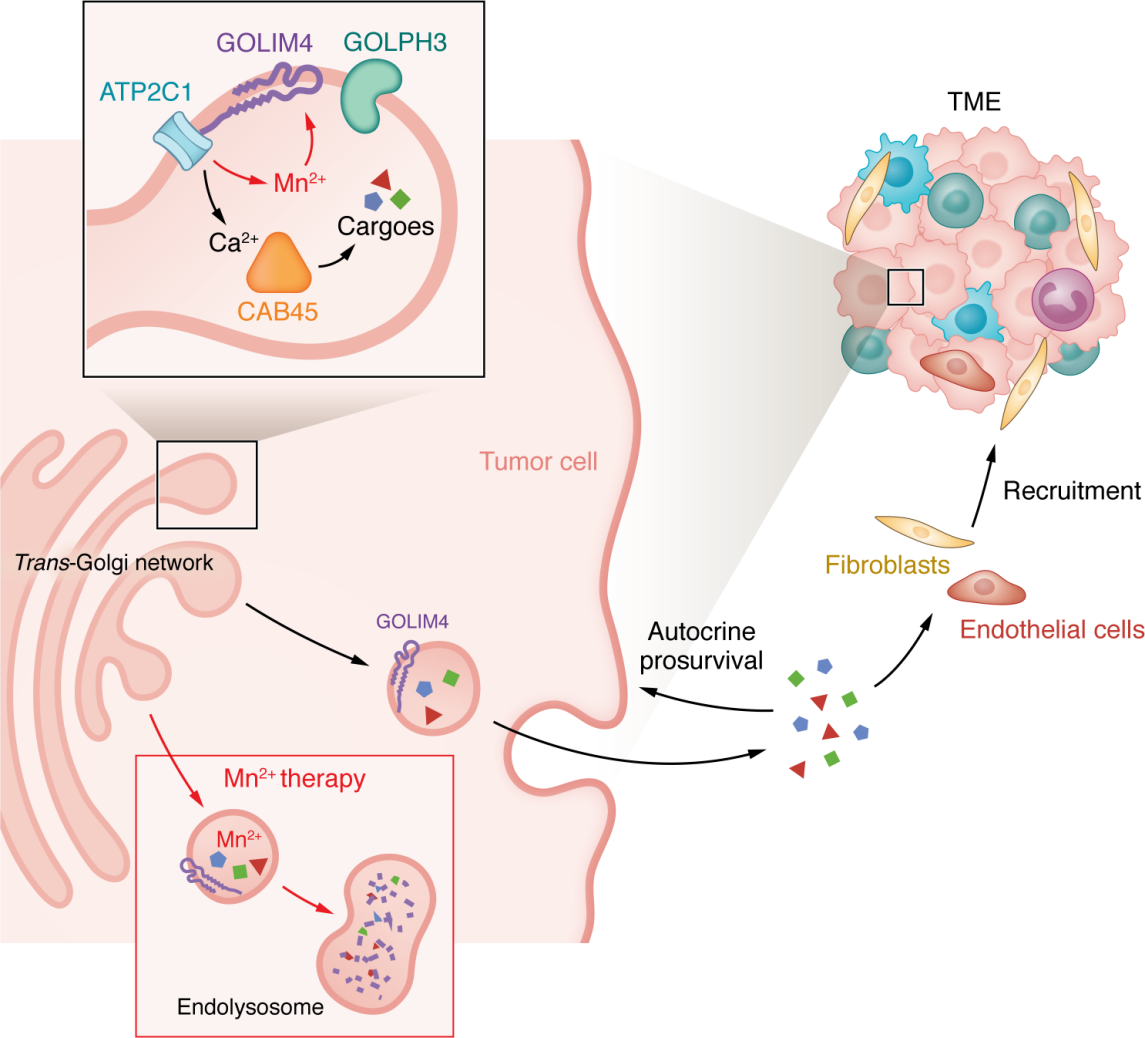
GOLIM4 regulates a biosynthetic secretory program in 3q-amplified tumors. GOLIM4, an integral Golgi membrane protein that is upregulated in 3q-amplified malignancies, interacts with the Ca^2+^/Mn^2+^ channel ATP2C1 and the vesicle-budding regulator GOLPH3. This interaction promotes secretion of cargoes via the *trans*-Golgi network and recruits fibroblasts and endothelial cells to the TME, thus coordinating a secretory program that promotes survival of 3q-amplified cancer cells. Administration of Mn^2+^ diverts GOLIM4 to the lysosome, leading to its degradation and reduction of the 3q-associated secretory program.

The elegant description of functional cooperativity between GOLIM4 and ATP2C1 was particularly interesting. ATP2C1, a Golgi-resident calcium/manganese (Ca^2+^/Mn^2+^) channel also present in the 3q amplicon, regulated various aspects of the biosynthetic secretory pathway. Notably, tumor-bearing mice treated with Mn^2+^ for 2 weeks showed reductions in tumor size and metastasis. The findings imply that GOLIM4-mediated tethering of ATP2C1 in the Golgi membrane results in Ca^2+^ entry to promote cargo sorting through the effector Ca-binding protein 45 kDa (CAB45) and suggest that this process is opposed by Mn^2+^, which enters the Golgi through ATP2C1 and binds GOLIM4 to target it for degradation in the lysosome (Figure 1). Thus, increasing Mn^2+^ levels abrogates GOLIM4-dependent secretory programs and reduces tumor progression (Figure 1). By exploring Mn^2+^ therapy in preclinical models, the authors open the door for targeting the whole secretory program in 3q-amplified malignancies, an endeavor worth exploring further.

The potential for targeting a particular hypersecretory phenotype as a whole, rather than focusing on specific proteins secreted by malignant cells, opens an opportunity for clinical impact. Indeed, cancer secretomes have previously been used as fishing grounds for therapeutic targets. It should be noted that these types of secretomes may act in dual capacities, having pro- and antitumorigenic functions. Indeed, this caveat is highlighted by reports that secretomes, such as the SASP, can promote tumor surveillance/clearance and tumor progression (9–12). This double-edged-sword property of proinflammatory and ECM-rich secretomes may be due to fine differences in the composition or quality of SASP-like secretomes that depend on biological context. Alternatively, and more likely, a pro- or antitumor outcome may be a matter of quantity. Thus, a secretome that, when produced in limited amounts, can recruit immune cells to clear tumors, may, when produced in larger quantities and for longer periods of time, lead to chronic inflammation, deposition of desmoplastic ECM, and tumor progression. This biological complexity must be considered when designing strategies for antisecretory therapies.

## Conclusions and future questions

The study by Tan et al. (13) is exciting because it demonstrates that Mn^2+^ can exert antitumor activity by suppressing the cancer hypersecretory phenotype. This indicates the possibility of a therapeutic regimen in which cancer cell secretion may be “dialed down” to reduce tumor promotion, while retaining some potential tumor-suppressive properties of the secretome of 3q-amplified tumors.

In addition to addressing the biological complexity incumbent in the double-edged-sword characteristics of proinflammatory secretomes, we feel that further preclinical investigations are needed before Mn^2+^ can be advanced as an anticancer therapy. Future studies should also consider the contribution of altered endosomal trafficking to metastasis in 3q-amplified tumors, given the presence of GOLIM4 in endosomes, its role in endosome-to-Golgi transport, and the ability of Mn^2+^ to subvert this function (15). Indeed, endosomal trafficking in tumor cells is key to their invasiveness and also facilitates their ability to prime metastatic niches. For instance, altered endosomal trafficking can change exosome composition to influence metastatic niche priming and subsequent metastasis (16, 17). Consistently, mice implanted with GOLIM4-depleted cells showed a marked reduction in metastasis in the work by Tan et al. (13).

Further experiments should also examine the role of GOLIM4 in the TME. Tan and authors emphasized autocrine aspects of GOLIM4-coordinated secretion. However, secretory programs, such as the one described by Tan et al. (13) that promoted cancer cell survival and activated the microenvironment, are often important in reprogramming of the stroma to support cancer cell growth and evasion of immunosurveillance. Determining the effects of the GOLIM4-driven secretome on stromal cells, and particularly on the immune system, will strengthen the case for preclinical evaluation of Mn^2+^ therapy for the treatment of 3q-amplified cancers. Next steps that explore the interaction of Mn^2+^ therapy with immunotherapies, which are now becoming the standard of care for many cancers, will provide a basis for clinical studies.

## Version 1 06/17/2024

Electronic publication
